# The complete chloroplast genome sequence of *Altingia excelsa*

**DOI:** 10.1080/23802359.2019.1710277

**Published:** 2020-01-14

**Authors:** Dejun Yang, Qiong Qiu, Linhong Xu, Yumei Xu, Yi Wang

**Affiliations:** a Institute of Tropical Forestry, Yunnan Academy of Forestry, Puwen, Yunnan, People’s Republic of China;; b Laboratory of Forest Plant Cultivation and Utilization, Yunnan Academy of Forestry, Kunming, Yunnan, People’s Republic of China

**Keywords:** *Altingia excelsa*, chloroplast, Illumina sequencing, phylogenetic analysis

## Abstract

The first complete chloroplast genome (cpDNA) sequence of *Altingia excelsa* was determined from Illumina HiSeq pair-end sequencing data in this study. The cpDNA is 160,861 bp in length, contains a large single copy region (LSC) of 89,126 bp and a small single copy region (SSC) of 19,011 bp, which were separated by a pair of inverted repeats (IR) regions of 26,362 bp each. The genome contains 127 genes, including 82 protein-coding genes, 8 ribosomal RNA genes, and 37 transfer RNA genes. Phylogenomic analysis showed that *A. excelsa* and *Liquidambar formosana* clustered in a clade in Saxifragales order.

*Altingia excelsa* is the species of the genus *Altingia* within the family Hamamelidaceae (Wang et al. 2007). It is distributed in the southeast and southwest of Yunnan and Motuo of Tibet in China, also found in India, Myanmar, Malaysia and Indonesia. Its bark and leaves contain aromatic oil, its trunk produces aromatic resin, and their twigs are edible (Kanjilal et al. 2003). It is a kind of high-quality timber for construction and plywood production (Wang et al. 2013). *Altingia excelsa* is one of the timber forest and short-term industrial raw material forest with development prospect in Yunnan hot area (Su et al. 2005). It is a widely used plant having great exploitation potentiality, however, there has been no genomic studies on *A. excelsa.*

Herein, we reported and characterized the complete *A. excelsa* plastid genome. The GenBank accession number is MN106247. One *A. excelsa* individual (specimen number: 201807062) was collected from Puwen, Yunnan Province of China (22°25′40′′ N, 101°6′56′′ E). The specimen is stored at Yunnan Academy of Forestry Herbarium, Kunming, China and the accession number is YAFH0012864. DNA was extracted from its fresh leaves using DNA Plantzol Reagent (Invitrogen, Carlsbad, CA).

Paired-end reads were sequenced by using Illumina HiSeq system (Illumina, San Diego, CA). In total, about 27.1 million high-quality clean reads were generated with adaptors trimmed. Aligning, assembly, and annotation were conducted by CLC de novo assembler (CLC Bio, Aarhus, Denmark), BLAST, GeSeq (Tillich et al. 2017), and GENEIOUS v 11.0.5 (Biomatters Ltd., Auckland, New Zealand). To confirm the phylogenetic position of *A. excelsa*, other 12 species of order Saxifragales from NCBI were aligned using MAFFT v.7 (Katoh and Standley 2013). The Auto algorithm in the MAFFT alignment software was used to align the 13 complete genome sequences and the G-INS-i algorithm was used to align the partial complex sequences. The maximum-likelihood (ML) bootstrap analysis was conducted using RAxML (Stamatakis 2006); bootstrap probability values were calculated from 1000 replicates. *Chloranthus spicatus* (EF380352) and *Buxus microphylla* (EF380351) were served as the out-group.

The complete *A. excelsa* plastid genome is a circular DNA molecule with the length of 160,861 bp, contains a large single copy region (LSC) of 89,126 bp and a small single copy region (SSC) of 19,011 bp, which were separated by a pair of inverted repeats (IR) regions of 26,362 bp each. The overall GC content of the whole genome is 37.9%, and the corresponding values of the LSC, SSC, and IR regions are 36.0, 32.2, and 43.0%, respectively. The plastid genome contained 127 genes, including 82 protein-coding genes, 8 ribosomal RNA genes, and 37 transfer RNA genes. Phylogenetic analysis showed that *A. excelsa* and *Liquidambar formosana* clustered in a unique clade in Saxifragales order (Figure 1). The determination of the complete plastid genome sequences provided new molecular data to illuminate the order Saxifragales evolution.

**Figure 1.**
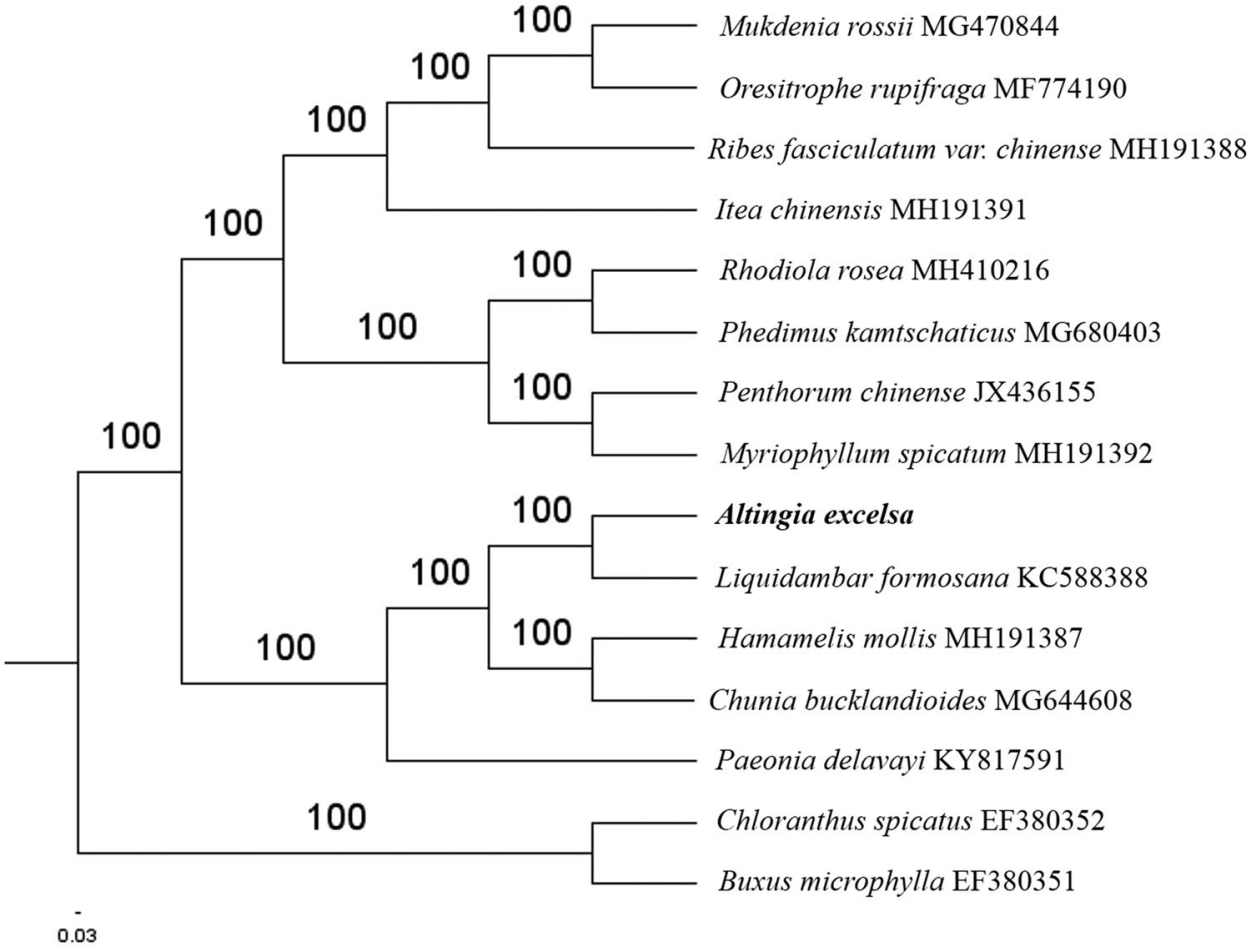
The maximum-likelihood tree based on the 13 chloroplast genomes of order *Saxifragales*. The bootstrap value based on 1000 replicates is shown on each node.
